# Development of an acceptable indigenous food diet for Pedi children under five years in early childhood development centers in rural Limpopo, South Africa

**DOI:** 10.1186/s13690-021-00743-9

**Published:** 2021-11-30

**Authors:** Gundo Nepfumbada, Tafadzwa Dzinamarira, Tivani Phosa Mashamba-Thompson

**Affiliations:** University of KwaZulu-Natal - Howard College Campus, Durban, South Africa

**Keywords:** Indigenous food diet; Early childhood development; Key stakeholder

## Abstract

**Background:**

The use of indigenous food (IF) such as green leafy vegetables and fruits in rural communities has been the primary source of their diet despite being replaced by food high in sugar and fats. South Africans are over-reliant on maize and should diversify their diets to include more indigenous fruits and vegetables to improve nutrition. Early Childhood Development (ECD) centers positively influence healthy eating among children under five years. This study aimed for ECD stakeholders to co-create an IF diet for children under five in ECD centers.

**Method:**

A sequential explanatory mixed-method design was employed. We conducted focus group discussions with stakeholders using the community-based participatory research (CBPR) approach and the nominal group technique ranking method to develop children's acceptable indigenous food diet. Data were analyzed using both qualitative and quantitative methods. We employed a thematic approach to analyze data using a Consolidated Framework for Implementation Research (intervention characteristics, inner setting, outer setting, individuals involved in implementation, and the implementation process. We used statistical analysis to analyze quantitative data collected through surveys.

**Results:**

Participants developed an IF diet. Participants were six ECD stakeholders (ECD managers, social workers, and dieticians) aged 34-52. Participants identified and voted for Ditokomane, Oranges, Mabele soft porridge, Dithotse, and Dinawa as components of an IF that are suitable and acceptable for children under five years as an IF diet appropriate and adequate for children under five years ECD centers will implement.

**Conclusion:**

Implementation of the developed IF diet can be considered an intervention towards achieving the United Nations Sustainable Development Goal 2 to end hunger, achieve food security and improve nutrition and sustainable agriculture. The study suggests that the IF diet could scale up the use of IF to fulfill dietary requirements for children under five years and preserve indigenous knowledge.

## Background

Indigenous foods (IF) are staple food sources for many people in the world [1]. Many rural communities produce and consume indigenous food sources in Africa to accomplish food security and improve nutrition. Indigenous people of rural South Africa have long relied on IF as part of their diet due to their nutritional, cultural, and economic importance for health [2]. The diet changed as a result of colonization [3]. The most common feeding practice for children in South Africa is mixed feeding, which impacts health and exposes them to the risk of malnutrition [4]. Edible plants proved the high nutritional value and played an essential role in preventing malnutrition [1]. Local harvested IF have great potential to improve food and nutrition security in Sub-Saharan Africa [5]. In Zimbabwe, poor households continue to rely on wild fruits and vegetables as alternatives to cultivated foods plants for dry season's meals [1].

Similarly, in Swaziland, IF are still essential and contribute to a more significant annual diet than retail food [1]. In rural areas, there may be multiple nutritional benefits of IF because they grow naturally and are readily available, accessible, and inexpensive [6, 7]. They are rich in dietary qualities, and they supplement food and nutritional security [7]. Furthermore, they contribute to more than 50% of vitamin A and zinc in children's overall diet [8]. According to the Nutrition Guidelines for Early Childhood Development (NGECD), early childhood development (ECD) is a good time for children to learn essential healthy eating [9]. However, poor nutrition, poverty, and lack of access to nutritious food threaten the full potential development of over 43% of children under five years, according to the United nation children's fund (UNICEF) [10]. Moreover, poor nutrition, early introduction to fatty and high-energy dense diets result in lower cognitive, language, and psychosocial outcomes of children [8, 9]. South African pediatric food-based guidelines recommend an adequate and balanced diet, meet nutritional requirements, and lowers the risk of non-communicable diseases [10].

ECD centers provide a valuable mechanism for delivering nutrition in needy communities [11, 12]. The Children's Act No.38 of 2005 obliged ECD centers to ensure daily nutritious meals for children. Optimal nutrition during childhood is critical to ensure optimal health, growth, and development, as documented in the NGECD [13]. Menu planning, providing nutritious meals, and establishing food gardens in line with Food-Based Dietary Guidelines (FBDG) are essential for nutritional and developmental outcomes [11]. In line with the FBDG, different colored, textured IF food should be offered to preschool children frequently [11]. However, the absence of policies promoting locally available IF remains a challenge that needs attention to scale up the use of IF [13].

South Africa has the most significant biodiversity in the world, its system is based only on few crops, and there is an over-reliance on maize [5]. Despite the availability of IF, consumption frequency in rural areas is currently shallow and declining [3]. Many factors have been reported which affect the consumption of IF in general, with several underutilized IF reported despite being a sustainable and nutritious food source [7, 14]. Sources of food in children's diet vary from one society to another obtained from their agricultural plots [15]. Lack of information on the nutritional value of IF remains a challenge in realizing their benefits and expanding their use and promotion [6]. Availability, preparation, role modeling, and caregivers' nutritious knowledge are possible barriers to a healthy diet [14]. This barrier has prompted the Western Cape initiative to train ECD practitioners to develop affordable, nutritious meals, preparation, and food gardens [11]. Both imperfect knowledge of the nutritional benefits of IF and the growing disinterest of the younger generation contribute to these food being underutilized [16]. In addition, the collapse of knowledge on preparation and conservation skills contributes to their underutilization [17]. A decline in agricultural activities, community, and household food gardens threatens both the availability and access of IF for a healthy diet [18].

The Department of Social Development policy of food subsidy was developed to ensure food access and availability in ECD centers [8]. However, their focus is on quantity, with little attention on ensuring dietary diversity and quality [9]. In South Africa, the national food and nutrition security policy advocates for nutrient-dense underutilized IF as a promotion strategy [14]. So far, little has been reported on the interventions to promote the success of the intervention and other new promotional interventions on the use of IF diet amongst children in ECD centers. As a follow-up study from an unpublished manuscript on the knowledge, availability, and use of IF in ECD centers, our study aims to develop an acceptable IF diet to promote the implementation of an intervention to encourage IF use. It is anticipated that the findings of this study will help guide ECD policymakers on the most appropriate multi-sectoral approach for promoting implementing IF diet interventions for children ECD centers in rural South Africa.

## Methods

### Study setting and design

We used community-based participatory research (CBPR) approach to develop the IF diet in the rural Fetakgomo-Tubatse Municipality, Sekhukhune district, Limpopo province. Sekhukhune is one of the five districts in Limpopo, with 53% of severely food insecure households. Fetakgomo-Tubatse is one of the four municipalities in the Sekhukhune district and is the biggest; it accounts for 42 % of the geographic area, with villages scattered throughout with over 2200 indigenous species. Lack of water and insufficient rainfall remains a significant concern. However, the majority continue to grow IF crops in their gardens as their primary food to provide a dietary requirement for a balanced intake. This area has limited access to running water and experiences low rainfall throughout the year. However, most community members grow indigenous food crops in the home gardens as their primary food to provide additional dietary requirements for balanced intake. Our previous unpublished work revealed that dinawa, lerotho, leotja, thepe, and magaba are locally available IF they can easily be accessible during the rainy season. Some are available in the dry season.

Focus group discussion (FGD) was employed, followed by a survey and expert review on a diet using the Nominal group technique (NGT) to rank votes. The use of the CBPR approach has helped identify the community's needs to address eating behaviors through an IF diet and identify available resources needed to develop the IF diet and promote its use.

### Sampling

The study was conducted with a sample of 9 people conveniently selected community members involved in three ECD centers as stakeholders, although only six attended the FGD. The participants comprise 6 Pedi, 1 Tsonga, 2 Venda speaking people, two males, and seven females. Most of the participants, 63%, were Christians belonging to different Christian churches. Only 18% belong to the Zionist church; for this study, stakeholders were categorized into ECD manager, social workers, and a dietician involved in the development and monitoring of the menu.

### Data collection

The researcher employed a sequential explanatory mixed-method design was for data collection. We collected qualitative and quantitative data from ECD stakeholders through FGD guided by a designed interview tool and a questionnaire with a pre-populated list of IF types. This was followed by an expert review of the proposed IF diet developed. The questionnaire addressed the sociodemographic characteristics of stakeholders (gender, age, years of working experience, and level of education). Nominal group technique (NGT) stage four of ranking the votes was employed [19]. Stakeholders rated the IF prioritized to develop the diet; ranking was done from highest to lowest priority on a scale of 5-1; we calculated the number of votes per IF to get the percentage.

Before conducting focus group discussion, we held a planning meeting with the research team and the stakeholders. We outlined the purpose of the planning meeting to attendees. The planning meeting addressed the aim of the research and the entire data collection process. Following obtaining informed consent from participants, CBPR approach was explained to equip participants with knowledge of the CBPR approach. FGD and survey methods that were used for the development of the IF diet for children were outlined. Participants were given time to seek clarity and make suggestions.

### Focus group discussion

We used FGD to collect qualitative data from the stakeholders as participants following the CBPR approach. An interview guide was designed following the review of some findings of our previous study on knowledge and the use of available IF in the same setting. The series of questions including stakeholders knowledge on the current use of IF, strategies that can be used to improve the use of IF among children, proper feeding practices for children under five, challenges and limitations hindering the use of IF to develop an acceptable IF diet implementation by children in selected ECD centers. The focus group was moderated by one of the stakeholders and conducted in Sepedi, which is the local language, and lasted for no more than 60 minutes. The first author (GN) took notes, and the discussion was audiotaped. The findings of the focus group informed the design of the questionnaire.

Quantitatively, a questionnaire with close-ended questions with a pre-populated list of IF's was administered to stakeholders to calculate the number of food items included in the IF diet. The pre-populated IF was obtained from the previously unpublished work we conducted and the focus group where stakeholders listed the different IF types based on favorability amongst the children, accessibility, acceptability, and availability. The survey was completed individually.

### Expert's review

Data collection was concluded by employing one of the CBPR principles of promoting collaborative and equitable partnership by recognizing participant's knowledge and expertise. Experts involving dieticians, social workers, and ECD managers reviewed the list of IF voted for to develop an IF diet that is suitable and acceptable for children under five in ECD centers. Their review informed a follow-up on providing knowledge of the research, what it entails, and the IF diet that will be developed for implementation. After being reviewed by experts, a developed IF diet was presented to the children participating in the research. Parents expressed interest in the diet and even suggested that they be given to use it at home.

### Data analysis

The tape-recorded focus group discussion was transcribed verbatim in Microsoft word 2018. Verbatim transcripts of the conversation with stakeholders were performed to ensure the validity of the interviews. Interviews were uploaded into NVIVO 12 software for analysis. A thematic approach to qualitative data analysis with a coding framework guided by Consolidated Framework for Implementation Research (intervention characteristics, inner setting, outer setting, characteristics of individuals involved in implementation, and the implementation process) was employed to analyze focus group discussion data. A framework-based thematic analysis was performed by GN and TD in parallel guided by Consolidated Framework for Implementation Research (CFIR) [19]. CFIR approach allows for identifying major domains affecting the implementation of the intervention [19, 20]. This approach enabled domains identified in advance in the CFIR to be explicitly and systematically considered in the analysis while also facilitating enough flexibility to detect and characterize issues that emerged from the transcripts [21].

Qualitative data were analyzed using an inductive coding technique. A coding frame was derived from the CFIR framework with IF availability, use, and acceptability codes. A hybrid approach was employed to incorporate different sets of themes that came from the interview against the CFIR, and other sets emerged from the transcripts [22]. Focus group transcripts were reviewed line by line using a basic thematic approach.

Quantitative questionnaire data was collated in a spreadsheet to analyze the level of agreement of stakeholders on the IF types to be included in the IF diet. Descriptive data analysis was carried out to obtain the association between dependent and independent variables. The t-test was used to determine the variances between variables. In addition, 95% CI estimates were used to examine associations.

NGT ranking process was employed to analyze quantitative data from the participants. Ranking their ideas on a scale of 1-5 was done by allocating votes to each concept. The overall priority score for each theme was then calculated. This was done by capturing ranking responses and calculating overall priority scores.

Sequential exploratory mixed-method data was integrated. Coded focus group data and quantitative data were organized into a framework. We adopted a case and theme-based approach to managing our data through summarization and synthesis. Codes documents were constructed systematically and integrated relevant data under each code to accommodate both focus group transcripts and statistical data for analyses. Key themes were identified and collated to gain integrated conclusions from our mixed-method study.

## Results

### Characteristics of study participants

In total, the FGD comprised six participants from 34-52**.** The attendance rate was 66.6% since nine participants were expected, and only six participated. Reasons for non-attendance include work commitment and urgent family responsibility. Characteristics of the participants are outlined in Table 1.

**Table 1:** Presentation of participants by age, gender, level of education and years of working experience in working with Early Childhood Development centers in Fetakgomo municipality

Participant ID#	Age	Gender	Level of education	Years of working experience
01	34	M	Postgraduate	7
02	52	F	Diploma	22
03	45	F	Diploma	15
04	48	F	Diploma	15
05	38	M	Postgraduate	10
06	36	F	Postgraduate	7

### Focus group discussion

Stakeholders in the focus group discussion expressed positive attitudes towards the IF diet for children in ECD centers. CFIR domains on intervention characteristics – intervention source, evidence strength and quality, relative advantage, adaptability, trialability, complexity, design quality, and packaging and cost were thematic issues from the framework analysis of interview data. Framework analysis of the FGD transcript using the Consolidated framework for implementation research is presented in Table 2 on pages 16-18 of the manuscript.

**Table 2:** Framework analysis of the Focus group discussion transcript using the Consolidated framework for implementation research

Intervention Characteristics	
a) **Intervention source:** Stakeholders shared their views on the current diet of children and knowledge on types of indigenous food they perceived are suitable, accessible and available and should be included in the diet and address the poor eating habits. Stakeholders also identified the need for parents to be involved. *"Children are not fed properly the way they should be; the food that is being introduced are not good for their growth and development and don't include fruits and vegetables."* *"Dinawa being grown from the gardens."* *"Sweet potato can be included as part of food, potatoes, beetroot, leaves of sweet potatoes"* *"To add to the list, peanuts can be favorable either dried or boiled"* "*The good strategy is to limit the amount of food, some kids have big stomach and we think they are healthy, kids need to be taught how to eat/eating behavior as they don't differentiate, they just want to eat what is available"* "*Parents should be fully involved to assist the children healthy eating"*	
b) **Evidence Strength and Quality**: Stakeholders shared their knowledge on the preparation of IF for improved children's health and development. Stakeholders are of the perception that some IF can be cooked alone and some can be mixed together to make one dish. "*cooking styles makes food more favorable"* "*Dithlodi mixed with maize and make semothwane, they are small nice things we can cook as if its porridge and eat"* "*lerotse, we can do jam and spread on bread, they are always available depending on water"* *"Is like we can go back to the times of our grandparents, they were never sick, they ate food fresh from the ground, maize meal was processed from home without any chemical unlike now. I think the use of this food can contribute to the decline in diseases"*	
c) **Relative advantage**: Stakeholders perceived the IF diet to have a relative advantage over the current feeding practices currently implemented in ECD centers. *"The menu used in ECD centers should really need change and food should be strictly nutritious with inclusion of fruits and vegetables daily"* *"Good feeding practices involve teaching children quality of food than quantity as parents is interested in giving them more food"* *"Fatty foods must be avoided at all times"* "*Children should not eat IF only at school they must also eat at home so that they can develop well"*	
d) **Adaptability**: The stakeholders were of the perception that the IF diet would be fairly adaptable to meet children's needs at the ECD centers. Stakeholders highlighted the need for self-motivated actions that were attainable with as little effort as a center starting up a garden as a source for important diet constituents. *"I support the idea, lets change the menu, we must go back to the indigenous food diet, eat food like dikgobe, kgodu and homemade mageu"* *"We need to have gardens where we can grow our own food such as dinawa, spinach, this can help children to even see food from the soil before they are prepared, they can eat them in different ways. They will also know them from the soil not shops"*	
e) **Trialability:** Stakeholders expressed the need for early introduction of IF and selection of IF suitable for the children's health and development. *"Availability and accessibility of other IF may can be a problem"* "*Involves the introduction of food that is in line with the age of the children and food that will benefit their health outcomes and good development"* "*Fatty foods must be avoided at all times"*	
f) **Complexity**: Stakeholders expressed concern on some logistical challenges that may need to be overcome for successful implementation of the IF diet. Water scarcity emerged as one of those challenges. Further, stakeholders expressed the need to have parents fully on board with the program to ensure success. *"Water scarcity, parents not wanting their children to be fed"* *"Food is not treating their kids well. Parents should be fully involved to assist the children healthy eating."* *"Children must be able to see this food, if we say this is pumpkin, vegetables they must see them so that they can know them. Now we are buying them such as spinach due to water shortage. The knowledge of IF has disappeared*."	
g) **Design Quality and Packaging:** Stakeholders expressed the need to consider the quality of food than quantity as a good feeding practice for children. Early introduction of IF continue to adulthood. "*Good feeding practice involves teaching children quality of food rather than quantity as parents are interested in feeding them more food"* *"We must change the introduction of genetically modified food from early age and give them proper nutritious food such as fruits and vegetables"* "*For better health on children, the other method is to reduce the fatty foods, children start eating fatty foods from early childhood and continue to their adulthood, at the end they have diseases so we must avoid them"* "*We must teach children to eat fruits and vegetables at the ECD centers because they spend more time there"* *"IF makes people healthy, food of these days is no longer fresh due to injections"*	
h) **Cost**: Costs of the IF in the diet was raised as an important barrier to sustainability of the IF diet. Stakeholders raised concerns over other centers not being able to meet the costs of the requisite food products *"Other ECD centers may not follow the menu regularly if they fail to access the food, some may not have money to purchase them"*	

#### Ranking of priorities

Stakeholders identified a list of 16 IF types to be included in the IF diet. The ranking was done from highest to lowest priority using the rating score of 5-1. In this context, priority refers to the food voted most important for inclusion in the diet. Ditokomane, Oranges, and Mabele soft porridge scored the highest rating, porridge, mochaina, and lerotse scored the lowest ratings. The ranking results are presented in the Table 3.

**Table 3:** Ranking results in descending order of Indigenous food voted for by stakeholders

Types of IFS	Votes scores					Percentage of votes
	5	4	3	2	1	
Ditokomane (ground nuts)	6	0	0	0	0	38
Orange	6	0	0	0	0	38
Mabele soft porridge (grain sorghum)	5	1	0	0	0	36
Dithotse (Roasted pumpkin seeds)	4	2	0	0	0	35
Dinawa (beans)	4	2	0	0	0	35
Banana	4	2	0	0	0	35
Sweet potatoes	4	2	0	0	0	35
Spinach	4	1	1	0	0	34
Beetroot	4	1	1	0	0	34
Kgodu (vegetables soup)	3	2	1	0	0	26
Samp	3	2	1	0	0	26
Magaba (African potato)	2	3	0	1	0	24
Semperiane (maize, beans, seeds)	3	1	1	1	0	24
Porridge	3	1	1	0	1	23
Lerotse (watermelon)	0	2	2	3	0	20
Mochaina	1	1	2	2	0	19

### Developed IF diet

Experts used prioritized IF to develop co-create an IF diet menu for children. The following factors were considered before finalization: the frequency of use for each IF type suggested type of meal and preparation method. Expert's review is summarized in Table 4 below.

**Table 4:** Indigenous food diet menu for children under five years in Fetakgomo municipality

Indigenous Food	Frequency of use in a week	Type of meal	Preparation and serving
Ditokomane (ground nuts)	Twice a week	Lunch Afternoon Snack	Cooked mixed with samp Fried
Orange	Three times a week	Snack	Raw
Mabele soft porridge (grain sorghum)	Three times a week	Breakfast	Cooked and served with milk
Dithotse (roasted pumpkin seeds)	Once a week	Afternoon snack	Dried and serve
Dinawa (beans)	Twice a week	Lunch	Cooked alone or mixed with samp
Banana	Once a week	Snack	Raw
Sweet potatoes	Twice a week	Breakfast Afternoon snack	Mashed with milk Boil and serve
Spinach	Once a week	Lunch	Boiled with carrots served with porridge
Beetroot	Once a week	Lunch	Boiled served with samp
Kgodu (vegetable soup)	Once a week	Lunch	Cooked and served alone
Samp	Once a week	Lunch	Cooked mixed with beans

## Discussion

This study presents the results of a developed IF diet for children under five years in ECD centers, in line with food sources available in their area. Stakeholders shared their knowledge and perceptions of IF types to develop an IF diet for implementation in ECD centers. Experts perceived it suitable to address poor eating habits to improve the health and nutritional outcomes of children. This diet will help achieve Sustainable Development Goals (SDG) 3 and 4 that promote improved nutrition and ensure healthy lifestyles for all ages [23]. Participants designed a menu with fruits, vegetables, and mixed dishes in line with the South African FBDG [10]. Our findings reveal that stakeholders perceived an IF diet as essential in achieving the 2030 agenda to end malnutrition, stunting, and wasting for children under five years. An IF diet menu was developed considering critical factors such as frequency of use of the prioritized IF, the type of meal, how they should be prepared and served.

Furthermore, stakeholders developed the IF diet with the types of food suitable and acceptable for consumption for children under five years. IF such as ditokomane, oranges, mabele soft porridge, dinawa were prioritized for inclusion in the menu. The prioritized food is to be served twice a week, whereas the least prioritized food can be served once a week. Stakeholders expressed the need to consider IF preparation, the quality of IF, and the feeding practices during the diet implementation. In our study, stakeholders perceived using an IF diet as a strategy to address poor eating habits at an early age. This finding is backed by a report on IF and their contribution to nutritional requirements, which states that IF plays a significant role in enhancing quality diets [24]. Stakeholders perceived that IF could be cooked alone and mixed to make one dish. These findings support a previous study that highlights that combining fruits and vegetables has more potential benefits than single fruits and vegetables [25].

Furthermore, sufficient intake of fruits and vegetables has reduced the risk of many non-communicable diseases [25]. Challenges of accessibility, lack of interest were reported that might affect the implementation of IF diet were reported elsewhere [8, 26]. Similar challenges were reported from a study conducted on the role of fruits and vegetables in delivering healthy diets challenges on accessibility and acceptability of IF [26]. Stakeholders expressed the need for early introduction of IF diet for children's health and development. However, a study on rural parent support on child health behavior reveals that children are fed food that does not support their growing bodies and brains [27]. Early exposure to unhealthy diets low in nutrient-dense food negatively impacts children's cognitive development [28].

The developed IF diet in the current study includes fruits, vegetables, and mixed dishes; stakeholders perceive it as an initiative to promote healthy eating. This is similar to the diet of the Boka children of Cameroon, whose diet has more tubers, fruits, and green leafy vegetables [15]. In addition, FBDG recommends that different colored, textured, and tasting fruits and vegetables, both fresh and cooked, be frequently offered to children [10]. Stakeholders emphasized the frequent consumption of both fruits and vegetables such as oranges, bananas, spinach, and beetroot daily. Okop,2019 reports that adequate intake of fruits and vegetables is considered essential for optimum growth [29]. However, in the current study, amounts of IF to be consumed were not indicated. This is contrary to the survey by Ramsay, 2017 which reports that children should be fed specific amounts of fruits and vegetables to optimize growth [30].

Nutrition interventions targeting ECD centers need to be strengthened to promote and encourage the early introduction of an IF diet for healthy eating for improved nutritional outcomes. Future studies should focus on the nutritional values of IF to ensure that the diet addresses the nutritional status of children under five years. The government should strengthen collaborations between the Department of Agriculture, health, and social development to ensure accessibility, acceptability, and affordability and scale-up IF's use in ECD centers.

The study has both strengths and limitations. A necessary strength was using FGD and NGT to gather data on perceptions, accessibility, availability, and preferability of IF and rate them for inclusion in the diet. This method allows replicating the study with other communities exhibiting the same and/or different cultures in South Africa and comparing locations and cultures. Another strength of this study is that all stakeholders involved in developing an IF diet work with ECD centers and children under five years. The participants comprised of different age groups and gender shared different perceptions regarding the suitable and acceptable diet for children under five. The involvement of parents in the planning process could promote the use of IF even at home as they were happy with the diet. However, the study was limited to small sample size and conducted in three ECD centers in different villages but under one district/municipality, which may not be the entire province and/or country. Some of the invited stakeholders did not show up, thus limiting the knowledge and expertise gathered. Stakeholders from other settings were omitted and thus limiting knowledge and expertise from other locations towards the study findings.

## Conclusion

The current findings demonstrated how stakeholders, parents/caregivers' perceptions, and acceptability of IF diet influence consumption of IF in the South African context. Stakeholders perceived frequent use of different types of IF as essential for optimum health and growth of children. Parents should be more involved and equipped with nutrition education to promote the early introduction of an IF diet both at ECD and at home. The importance of moral regeneration to conserve and prevent loss of indigenous knowledge. There is a need for further research with stakeholders from different settings to increase the generalizability of the study results.

## Data Availability

The data used in this study are available from the corresponding author on reasonable request. Data users may, however, be required to seek permission from the original data providers.

## Abbreviations

IF: Indigenous food
ECD: Early childhood development
CFIR: Consolidated framework for implementation research
CBPR: Community-based participatory research
NGECD: Nutrition guidelines for early childhood development
UNICEF: United nation children's fund
FBDG: Food based dietary guidelines
NGT: Nominal group technique
